# Sphingolipid Metabolism in the Pathogenesis of Hashimoto’s Thyroiditis

**DOI:** 10.3390/ijms262110674

**Published:** 2025-11-02

**Authors:** Jialiang Huang, Zeping Chen, Yijue Wang, Chuyu Shang, Yue Feng

**Affiliations:** School of Acupuncture and Massage, Chengdu University of Traditional Chinese Medicine, Chengdu 611137, China

**Keywords:** Hashimoto’s thyroiditis, sphingolipid metabolism, autoimmunity, immune cell infiltration, thyroid fibrosis, S1P

## Abstract

Hashimoto’s thyroiditis (HT) is the most common autoimmune thyroid disorder, characterized by progressive lymphocytic infiltration, follicular destruction, tissue fibrosis, and an elevated risk of thyroid carcinoma. While the precise mechanisms underlying HT remain incompletely defined, emerging evidence implicates dysregulated sphingolipid (SPL) metabolism, particularly the sphingosine-1-phosphate (S1P) signaling axis, as a central contributor to disease pathogenesis. S1P, a bioactive lipid mediator, integrates metabolic and immunological cues to regulate immune cell trafficking, cytokine production, apoptosis, and fibroblast activation. Aberrant activation of the sphingosine kinase (SPHK)/sphingosine-1-phosphate (S1P)/S1P receptor (S1PR) pathway has been linked to persistent T helper 1 (Th1) cell recruitment, signal transducer and activator of transcription 3 (STAT3)-mediated immune polarization, epithelial–mesenchymal transition, extracellular matrix remodeling, and the establishment of a chronic inflammatory and fibrotic microenvironment. Moreover, S1P signaling may foster a pro-tumorigenic niche, providing a mechanistic explanation for the strong epidemiological association between HT and papillary thyroid carcinoma. This review summarizes current insights into the role of SPL metabolism in HT, highlighting its potential as a mechanistic link between autoimmunity, fibrosis, and carcinogenesis.

## 1. Introduction

Hashimoto’s thyroiditis (HT) is the most prevalent autoimmune thyroid disorder, with a steadily rising global incidence that underscores its growing public health significance [1,2]. Despite extensive investigation, the pathogenesis of HT remains incompletely understood. Current evidence implicates a multifactorial etiology involving genetic susceptibility, selenium deficiency, and exposure to immunomodulatory agents as key contributors to disease onset and progression [3,4]. Sphingolipids (SPLs), fundamental structural components of cellular membranes, play critical roles in maintaining membrane integrity and functionality [5,6,7]. “SPL metabolism” encompasses the comprehensive network of biosynthetic, catabolic, and interconversion pathways that govern the production and turnover of all SPL species [8,9]. Within this metabolic framework, the S1P pathway represents a distinct and functionally critical branch. S1P, a potent bioactive lipid mediator generated from the catabolism of membrane-bound SPLs, orchestrates a wide array of biological processes [10,11].

The S1P pathway includes the classical SPHK/S1P/S1PR axis in which sphingosine kinases SPHK1 and SPHK2 act as rate-limiting enzymes controlling S1P homeostasis [12,13,14]. Following its generation, S1P undergoes extracellular export and mediates diverse biological effects through five specific G protein-coupled receptors (S1PR1–5), thereby modulating immune cell trafficking, inflammatory responses, and cellular fate decisions [15,16]. Recent studies suggest the pathological relevance of SPL dysregulation in HT. Notably, aberrant SPL metabolism has been associated with severe lymphocytic infiltration in HT, indicating its potential role in pathogenic immune cell recruitment [17]. Moreover, S1P levels have been shown to rise in response to membrane damage, a pathological feature consistently observed in HT thyroid follicles [18]. Although elevated S1P has been detected in both HT patients and animal models, its precise mechanistic contribution to thyroid follicular cell injury remains unclear. This review systematically summarizes current insights linking disruptions in SPL metabolism to the immunopathogenesis of HT, providing theoretical foundation for the development of SPL-based interventions in the treatment of HT.

## 2. Pathophysiological Overview of HT

HT is an organ-specific autoimmune disorder marked by the presence of thyroid-specific autoantibodies, dense lymphocytic infiltration, progressive tissue fibrosis, and eventual architectural disruption of the thyroid gland, culminating in hypothyroidism [19]. Mounting evidence implicates infiltrating B and T lymphocytes as central mediators in HT pathogenesis, with their accumulation in thyroid tissue serving as a histological hallmark of disease progression [20,21]. Through the release of pro-inflammatory cytokines and chemokines, these immune cell populations orchestrate a sustained autoimmune response that drives thyroidal injury and dysfunction [22]. Among the cytokines involved, tumor necrosis factor-α (TNF-α), interferon-γ (IFN-γ), and interleukin-1β (IL-1β) have been identified as key effector molecules contributing to thyroid destruction via multiple interrelated mechanisms [3,23,24]. Notably, IL-1β acts as a potent immunostimulatory cytokine by enhancing CD40 ligand (CD40L)-mediated activation of dendritic cells, promoting the antigen-dependent expansion of naïve CD4^+^ T cells and facilitating their differentiation into T helper 17 (Th17) effector subsets. Additionally, IL-1β has been shown to induce the expression of Fas on thyrocytes, a critical component of the Fas/Fas ligand (FasL) apoptotic signaling cascade [25,26,27], which is widely recognized as a principal mediator of programmed cell death [28]. Fas-dependent apoptosis is thus postulated to constitute a major effector mechanism driving thyrocyte loss and the development of hypothyroidism in HT [29]. Supporting this, comparative histopathological studies have demonstrated significantly elevated Fas expression in thyroid tissues of HT patients relative to those with simple goiter, implicating IL-1β–driven Fas upregulation as a pivotal trigger of immune-mediated thyrocyte apoptosis [30]. Despite extensive investigation since HT was first described, the molecular mechanisms underlying thyroid tissue destruction remain incompletely understood. Recently, dysregulated SPL metabolism has emerged as a potential contributor to HT pathophysiology. Aberrant SPL profiles have been identified in HT patients, suggesting a mechanistic role for lipid signaling perturbations in modulating immune responses and thyroidal inflammation [31]. These findings implicate a previously underexplored dimension of HT pathogenesis, warranting further investigation into this novel mechanistic pathway.

## 3. Roles of Sphingolipid Metabolism and S1P Signaling in Immune Regulation and Autoimmunity

### 3.1. Sphingolipid Metabolic Pathways

SPLs constitute a vital class of membrane lipids that play fundamental roles in maintaining cellular structural integrity and regulating critical biological processes [32]. As essential components of cellular organelles and plasma membranes, SPLs participate in a sophisticated metabolic network centered around ceramide (Cer), which serves as the pivotal hub for both biosynthetic and catabolic pathways [33]. The SPL family encompasses several biologically active molecules, including Cer, S1P, sphingosine (Sph), sphingomyelin, and glycosphingolipids, each of which is implicated in the regulation of key cellular functions such as proliferation, differentiation, senescence, and programmed cell death [34]. Ceramide biosynthesis occurs through three major routes. First, the de novo pathway is initiated by serine palmitoyltransferase-mediated condensation of serine and palmitoyl- CoA [35]. Second, the sphingomyelinase pathway involves hydrolysis of sphingomyelin. Third, S1P is dephosphorylated to sphingosine and subsequently re-acylated to regenerate ceramide in a specific salvage pathway [36,37]. A biologically consequential concept emerging from these interrelated pathways is the “sphingolipid rheostat”, a homeostatic balance between the pro-apoptotic lipids Cer and Sph, and the pro-survival lipid S1P [38,39]. This metabolic equilibrium plays a pivotal role not only in fundamental cellular fate decisions but also in diverse pathological processes [40,41], including autoimmune disorders such as HT [31,42], where dysregulation of SPL metabolism contribute to disease pathogenesis through modulation of immune responses and thyrocyte viability.

### 3.2. The Pivotal Role of Sphingosine-1-Phosphate in Sphingolipid Metabolism

S1P is a critical bioactive lipid mediator derived from the catabolism of membrane SPLs. Following its intracellular biosynthesis, S1P is actively exported into the plasma and lymphatic compartments, where it orchestrates a diverse array of cellular processes including proliferation, differentiation, and migration [43,44]. The synthesis and homeostasis of S1P are tightly regulated by two isoforms of sphingosine kinases, SPHK1 and SPHK2, which exhibit distinct subcellular localization and non-redundant biological functions [45,46]. SPHK1 is predominantly localized in the cytosol and translocates to the plasma membrane upon stimulation, a prerequisite for its enzymatic activation [47]. Elevated SPHK1 expression and consequent intracellular S1P accumulation have been implicated in enhanced proliferation and cell survival [48]. In contrast, SPHK2, which localizes primarily to the nucleus, has been associated with pro-apoptotic signaling [49]. Extracellular export of S1P is mediated by two specialized transporter systems, members of the ATP-binding cassette (ABC) transporter family and the spinster homolog 2 (SPNS2) protein [50,51]. Upon release into the extracellular milieu, S1P engages a family of five G protein–coupled receptors (S1PR1–S1PR5), whose expression is cell- and tissue-specific [52]. These receptors initiate downstream signaling cascades, including the PI3K and ERK1/2 pathways, which modulate essential cellular functions such as cytokine secretion, expression of adhesion molecules, and regulation of proliferation, migration, and apoptosis [53,54]. Thus, the spatially and temporally coordinated regulation of S1P synthesis, secretion, and receptor activation forms an integrated signaling network that links metabolic and extracellular cues to orchestrate complex physiological and pathological responses [55,56].

### 3.3. The Association Between Sphingosine-1-Phosphate and Autoimmune Diseases

Accumulating evidence implicates S1P signaling as a pivotal regulator in the immunopathogenesis of autoimmune disorders including multiple sclerosis (MS), rheumatoid arthritis (RA), and systemic lupus erythematosus (SLE) [57,58,59]. In RA, enhanced synovial S1PR1 expression promotes fibroblast-like synoviocyte proliferation, exacerbating joint destruction [60,61]. Functional studies reveal that S1P–S1PR1 signaling fosters synoviocyte proliferation, contributing to joint pathology. Notably, pharmacological blockade of SPHK using *N*,*N*-dimethylsphingosine substantially reduces circulating levels of IL-6, TNF-α, and IFN-γ in RA patients, signifying a suppression of systemic inflammatory responses [62]. Supporting these findings, SPHK1-deficient murine models exhibit attenuated disease activity and decreased inflammation-related morbidity, reinforcing the mechanistic role of S1P signaling in orchestrating pro-inflammatory pathways in RA [63]. In MS, phosphoproteomic analyses of demyelinated brain lesions revealed heightened phosphorylation of S1PR1, which correlated with exacerbated neuroinflammation [64]. Mechanistically, activated S1PR1 potentiates IL-6–dependent STAT3 signaling, thereby promoting Th17 cell polarization and amplifying autoimmune-mediated neuroinflammation [65]. Elevated SPHK1 and S1PR1 expression in patients with primary Sjögren’s syndrome and in models of autoimmune liver injury further illustrates the relevance of the S1P metabolic pathway across autoimmune phenotypes [15,66].

FTY720 (fingolimod), a functional antagonist of S1P receptors, has emerged as a clinically relevant immunomodulator targeting the S1P pathway [67]. In MS, fingolimod has demonstrated therapeutic efficacy, not only dampening neuroinflammation but also contributing to remyelination through direct regulatory actions on central nervous system (CNS) cell populations [68]. Preclinical evidence extends its therapeutic potential to experimental autoimmune myasthenia gravis and thyroiditis models of HT [69]. Moreover, FTY720 suppresses the fibrotic markers transforming growth factor-β (TGF-β) and α-SMA) across multiple organ fibrosis models [70,71]. However, concerns regarding cardiovascular toxicity and infection risk due to broad immunosuppression necessitate cautious clinical application [68,72]. Continued investigation of the S1P pathway dynamics offers promising avenues for precision immunomodulation in diverse autoimmune settings [73,74].

## 4. Sphingolipid Metabolism and Its Relevance to Hashimoto’s Thyroiditis

### 4.1. Association Between Sphingosine Kinase and Thyroid Follicular Membrane Disruption in Hashimoto’s Thyroiditis

Recent studies have demonstrated substantial dysregulation of SPL metabolism in both patients with HT and experimental autoimmune thyroiditis models, with a particular emphasis on the aberrant activation of the SPHK/S1P/S1PR signaling axis [18]. This dysregulation is characterized by upregulated expression of SPHK, S1P, and its receptors S1PRs, a profile consistently observed in HT pathology. The inflammatory microenvironment of HT appears to be a principal driver of this metabolic reprogramming, wherein pro-inflammatory cytokines such as TNF-α, IL-1β, TGF-β, and vascular endothelial growth factor (VEGF) potently induce SPHK1 expression and enzymatic activity [75,76,77,78]. Mechanistic insights have demonstrated that TNF-α directly activates SPHK1 through ERK1-dependent phosphorylation events [79]. Elevated concentrations of TNF-α and IL-1β have been consistently reported in the context of autoimmune thyroid diseases [80]. Notably, both cytokines not only enhance SPHK1 activity but concurrently promote transcriptional upregulation of Fas in thyroid follicular cells (thyrocytes) via the NF-κB and AP-1 signaling pathways [81,82,83,84]. This convergence creates a pathogenic nexus whereby SPHK1-derived S1P signaling intersects with Fas/FasL-mediated apoptotic cascades [85].

**Figure 1:**
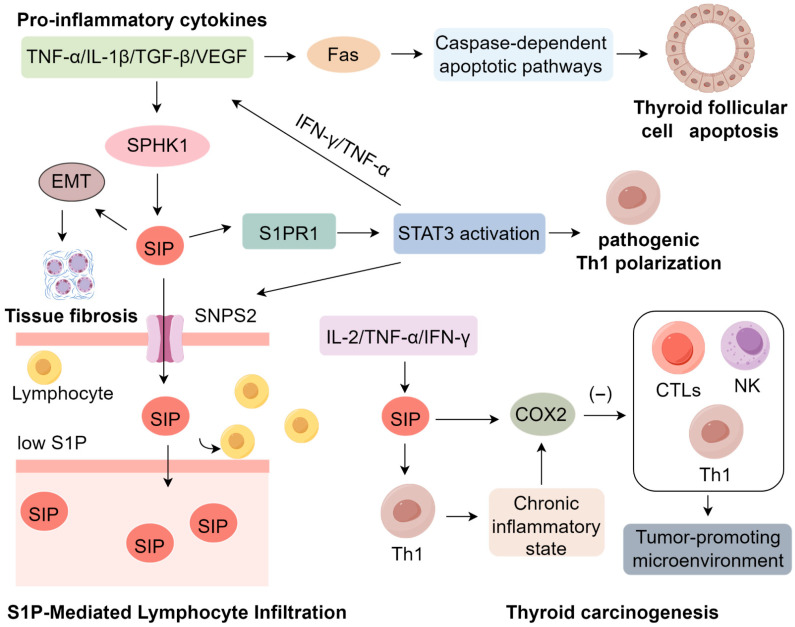
Mechanisms of S1P Signaling in Inducing Hashimoto’s Thyroiditis.

S1P functions as a crucial modulator of apoptosis by regulating key downstream effectors such as caspase-3 [86]. Elevated intracellular S1P concentrations facilitate the assembly of the death-inducing signaling complex (DISC) at the Fas receptor, thereby sensitizing thyrocytes to Fas-triggered apoptosis under inflammatory conditions [87,88]. Furthermore, engagement of S1P with its receptor S1PR1 activates the JNK signaling pathway, which synergistically amplifies Fas-mediated pro-apoptotic responses [89,90]. This mechanistic interplay suggests that IL-1β-induced SPHK activation, in concert with Fas upregulation, potentiates thyrocyte apoptosis, accelerating autoimmune-driven follicular destruction in HT [18,91,92]. Collectively, these findings position aberrant SPL metabolism, particularly dysregulated S1P signaling, as a central pathogenic mechanism underpinning the structural disintegration of thyroid follicles in HT. The SPHK/S1P/S1PR axis thereby emerges as a critical molecular link between chronic autoimmune inflammation and progressive thyroid tissue injury [44], underscoring its potential as a therapeutic target in autoimmune thyroid disorders (Figure 1).

### 4.2. Sphingosine-1-Phosphate-Mediated Lymphocyte Infiltration and Immune Dysregulation in the Pathogenesis of Hashimoto’s Thyroiditis

The characteristic lymphocytic infiltration observed in HT has been mechanistically linked to aberrant S1P signaling, which orchestrates both lymphocyte trafficking and immune cell differentiation [93]. Within thyroid tissue, S1P is actively exported via the SPNS2 transporter, establishing a concentration gradient characterized by low levels in secondary lymphoid organs and elevated levels in the peripheral circulation [94]. This chemotactic axis facilitates lymphocyte egress and subsequent infiltration into the thyroid gland [93,95]. In the autoimmune milieu of HT, this process is markedly amplified by upregulation of the S1P receptor 1 (S1PR1), as demonstrated in autoimmune thyroiditis models, where S1PR1 expression serves as a pivotal mediator of S1P-induced activation of signal transducer and activator of transcription 3 (STAT3) [18]. STAT3, a master regulator of CD4^+^ T cell lineage commitment, undergoes phosphorylation at Tyr705 and Ser727, enhancing its transcriptional activity and promoting the differentiation of pro-inflammatory Th1 cells [96]. However, the immunomodulatory effects of the S1P–S1PR1–STAT3 signaling cascade extend beyond Th1 polarization. This axis critically perturbs the equilibrium between Th17 cells and regulatory T cells (Tregs) [65,97], a skewing differentiation toward a pathogenic Th17 phenotype at the expense of Foxp3^+^ Treg development. This shift is driven by sustained STAT3 activation, which promotes IL-17 production while concurrently suppressing Treg-mediated immunoregulation [66,98]. The resultant Th17/Treg imbalance fosters a persistent pro-inflammatory microenvironment characterized by elevated IL-17 levels, increased neutrophil and macrophage recruitment, and breakdown of peripheral tolerance mechanisms [99,100,101]. In experimental models of HT, heightened Th17 activity is closely associated with exacerbated follicular cell damage and progressive thyroid gland destruction, whereas compromised Treg function contributes to unchecked autoimmune responses and disease acceleration [102,103]. Collectively, these findings underscore the central role of dysregulated S1P signaling in shaping the Th17/Treg axis and delineate a critical immunopathogenic mechanism driving the chronic inflammation and tissue injury characteristic of HT.

### 4.3. The Role of Sphingosine-1-Phosphate in Thyroid Tissue Fibrosis in Hashimoto’s Thyroiditis

Beyond lymphocytic infiltration, HT is frequently marked by progressive fibrosis, contributing to thyroid architectural remodeling and dysfunction [104]. The SPHK/S1P/S1PR axis has emerged as a central regulator of fibrotic remodeling across multiple organs including the heart, lungs, and kidneys [105,106]. Mechanistic studies have identified S1P as a key inducer of fibroblast-to-myofibroblast transdifferentiation, a pivotal step in extracellular matrix (ECM) deposition and scar formation [107]. During early stages of fibrosis, S1P synergizes with pro-inflammatory cytokines such as TNF-α, IFN-γ, TGF-β, and IL-6 to promote epithelial–mesenchymal transition (EMT), a reparative cellular reprogramming event that, when unresolved, drives pathological fibrosis and may even predispose cells to malignant transformation [108]. In renal fibrosis models, S1P robustly upregulates α-smooth muscle actin (α-SMA) in fibroblasts, facilitating their conversion into collagen-producing myofibroblasts [109,110,111]. Among S1P receptors, S1PR2 has been implicated in pro-fibrotic signaling via activation of the Rho/ROCK pathway, B cell niche organization, and amplification of cytokine cascades, whereas S1PR3 enhances myofibroblast differentiation and augments TGF-β/Smad signaling during fibrotic remodeling [112].

Recent studies in thyroid tissues from HT patients demonstrate that fibroblast populations expressing classical myofibroblast markers including α-SMA, vimentin, and fibronectin, and preferentially localize to regions with dense interstitial collagen deposition [113]. Concurrently, chronic exposure of thyrocytes to IL-6 and TGF-β induces EMT-like phenotypes via TGF-β/Smad-dependent transcriptional programs, implicating both resident fibroblasts and EMT-derived thyrocytes in the fibrotic matrix in this process [113,114,115]. Furthermore, sustained immune-stromal crosstalk—particularly mediated by IL-1β and TNF-α—perpetuates fibroblast activation, creating a pro-fibrotic microenvironment that, while mechanistically similar to fibrosis in other organs, exhibits thyroid-specific features [116,117]. Although elevated S1P and cytokine levels have been reported in HT patients, the precise mechanisms by which S1P orchestrates thyroid-specific fibrogenesis remain to be fully elucidated [118].

### 4.4. The Role of Sphingosine-1-Phosphate in HT-Associated Thyroid Carcinogenesis

Emerging evidence indicates a strong pathological link between HT and the development of thyroid malignancies, particularly that of papillary thyroid carcinoma (PTC). The persistent inflammatory microenvironment that defines HT is characterized by elevated levels of pro-inflammatory cytokines including IL-2, TNF-α, and IFN-γ, which collectively promote tumorigenesis through S1P-mediated pathways [119]. A pivotal study demonstrated that the Th1-skewed immune response in HT enhances SPHK1 expression, resulting in S1P overproduction [120]. This S1P-rich milieu not only sustains chronic inflammation but also upregulates cyclooxygenase-2 (COX2), an enzyme whose immunosuppressive properties suppress cytotoxic T lymphocytes (CTLs), Th1 cells, and natural killer (NK) cells—central mediators of anti-tumor immunity. Beyond immunosuppression, S1P–COX2 signaling intersects with oncogenic pathways [121]. Specifically, S1P activates PI3K/AKT cascade via S1PR1 and S1PR3 [122,123], thereby creating a permissive context for the proliferation and survival of genetically altered thyrocytes [122]. Simultaneously, elevated COX2 enhances prostaglandin E2 (PGE2) production, which in turn transactivates the EGFR–RAS–RAF–MEK–ERK pathway [124,125]. This crosstalk may synergize with BRAFV600E or RAS mutations, which are common driver events in PTC, leading to sustained MAPK activation [126,127]. The convergence of inflammatory metabolic signaling with BRAF-driven oncogenic cascades promotes thyrocyte dedifferentiation, genomic instability, and clonal expansion, thereby accelerating malignant transformation [128]. Concurrently, S1P-induced immunosuppression weakens immune surveillance, allowing mutated cells to evade detection while MAPK signaling drives tumor progression [129]. Notably, malignant thyrocytes themselves contribute to increased S1P levels, forming a feed-forward loop that links chronic inflammation to carcinogenesis [130]. Epidemiological studies reveal a striking 23% co-occurrence rate of HT and PTC in Chinese populations, with HT patients exhibiting higher rates of lymph node metastasis compared to PTC patients without HT [131]. Although these epidemiological and pathological associations are well established, the precise mechanistic contribution of S1P to this relationship remains incompletely defined [132,133]. Thus, the study of SPL metabolism represents a promising avenue for elucidating the mechanisms underpinning HT pathogenesis (Table S1).

## 5. Conclusions

HT is a complex autoimmune disorder marked by chronic lymphocytic infiltration, progressive destruction of thyroid follicles, tissue fibrosis, and an elevated risk of thyroid carcinogenesis. Despite decades of investigation, its underlying mechanisms remain incompletely defined. Recent advances highlight the central role of SPL metabolism—particularly the S1P signaling axis—in orchestrating key pathological events in HT. As a bioactive lipid messenger, S1P integrates metabolic, inflammatory, and immunological signals to regulate lymphocyte trafficking, pro-inflammatory cytokine release, thyrocyte apoptosis, and fibroblast activation. Aberrant activation of the SPHK/S1P/S1PR1 pathway drives persistent Th1 cell recruitment, STAT3-mediated immune polarization, EMT, and extracellular matrix remodeling, thereby amplifying autoimmune destruction and fibrotic progression. Moreover, the chronic inflammatory milieu maintained by S1P signaling may establish a pro-tumorigenic niche that predisposes HT patients to PTC.

Collectively, these observations position S1P dysregulation as a unifying mechanism linking immune activation, tissue injury, fibrogenesis, and oncogenesis in HT. Nevertheless, significant gaps remain regarding receptor subtype specificity, downstream signaling networks, and tissue-contextual effects. Addressing these gaps will be crucial for identifying biomarkers and devising targeted therapies aimed at modulating S1P signaling to preserve thyroid structure and limit disease progression. Future research should exploit single-cell RNA sequencing to delineate the cellular heterogeneity of HT lesions and organoid models to reproduce the thyroid immune microenvironment in vitro. In parallel, pharmacological strategies targeting SPHK/S1P/S1PR signaling, including selective SPHK inhibitors, may offer precision therapeutic approaches to prevent tissue damage and disease progression.

## Data Availability

No new data were created or analyzed in this study. Data sharing is not applicable to this article.

## Supplementary Materials

The following supporting information can be downloaded at: https://www.mdpi.com/article/10.3390/ijms262110674/s1.
