# Supplementary material for: Sphingolipid Metabolism in the Pathogenesis of Hashimoto’s Thyroiditis
Source: Int J Mol Sci. 2025 Nov 2;26(21):10674. doi: 10.3390/ijms262110674 (PMC12610107; doi:10.3390/ijms262110674)
Supplement: Supplementary file 1 [file ijms-26-10674-s001.zip › ijms-3883081-supplementary.pdf]

International Journal of *Molecular Sciences*

MDPI

Table S1. Roles of the SPHK/S1P/S1PR Axis in the Immunopathology of Hashimoto's Thyroiditis

Disease Process	S1P Axis Components	Signaling Pathways	Target Cell Types	Functional Outcomes	Impact
Thyrocyte apoptosis and follicular disruption	SPHK1, S1P, S1PR1, Fas/FasL	TNF-α-ERK1-SPHK1 (+)<br>NF-κB/AP-1- Fas (+)<br>S1PR1- JNK/caspase-3	Thyroid follicular epithelial cells (thyrocytes)	Promotes DISC assembly and Fas-mediated apoptosis under cytokine stress	Follicular destruction-hypothyroidism onset
Immune cell recruitment and amplification	SPNS2, S1P, S1PR1	S1P gradient across lymphoid–thyroid axis<br>S1PR1-STAT3 Tyr705/Ser727	$CD4^+$ T cells, macrophages, dendritic cells	Enhances lymphocyte trafficking<br>Induces Th1/Th17 polarization<br>Promotes IL-17–driven inflammation	Chronic inflammation<br>Immune amplification
Th17/Treg imbalance and immune tolerance breakdown	S1P, S1PR1	STAT3 sustained activation<br>Foxp3 expression (-)	Th17 cells, Tregs	Th17 (IL-17, RORγt) (+), Treg (Foxp3) (-)<br>Impaired immunosuppression<br>Exacerbated inflammation and autoimmunity	Immune dysregulation<br>Progressive tissue damage
Fibroblast activation and EMT-associated fibrosis	S1P, S1PR2, S1PR3	TGF-β/Smad<br>Rho/ROCK<br>IL-6/TNF-α synergy	Fibroblasts, EMT-derived thyrocytes	α-SMA, vimentin, fibronectin (-)<br>Myofibroblast differentiation<br>ECM accumulation and collagen deposition	Interstitial fibrosis<br>Thyroid architectural remodeling
Malignant transformation and tumor microenvironment formation	SPHK1, S1P, S1PR1/3, COX2, PGE2	PI3K/AKT<br>COX2-PGE2-EGFR–RAS–MAPK<br>S1P–COX2–PGE2 loop	Dedifferentiated thyrocytes, tumor-infiltrating immune cells	Cell survival and proliferation (-)<br>EGFR (-) signaling synergy with BRAFV600E<br>CTL/NK activity (-) - Immune evasion	HT-associated papillary thyroid carcinoma (PTC)
